# Identification and Morphological Characterization of Features of Circulating Cancer-Associated Macrophage-like Cells (CAMLs) in Endometrial Cancers

**DOI:** 10.3390/cancers14194577

**Published:** 2022-09-21

**Authors:** Raed Sulaiman, Pradip De, Jennifer C. Aske, Xiaoqian Lin, Adam Dale, Ethan Vaselaar, Cheryl Ageton, Kris Gaster, Luis Rojas Espaillat, David Starks, Nandini Dey

**Affiliations:** 1 Department of Pathology, Avera Cancer Institute, Sioux Falls, SD 57105, USA; 2 Translational Oncology Laboratory, Avera Research Institute, Sioux Falls, SD 57105, USA; 3 Department of Internal Medicine, University of South Dakota SSOM, Sioux Falls, SD 57069, USA; 4 Department of Research Oncology, Avera Cancer Institute, Sioux Falls, SD 57105, USA; 5 Outpatient Cancer Clinics, Avera Cancer Institute, Sioux Falls, SD 57105, USA; 6 Department of Gynecologic Oncology, Avera Cancer Institute, Sioux Falls, SD 57105, USA

**Keywords:** CAMLs, CTC, triple immuno-fluorescence, endometrial cancers, liquid biopsy

## Abstract

**Simple Summary:**

Circulating cells in the blood of cancer patients with solid tumors are known to provide clinically relevant information about treatment response, tumor progression, metastasis, and the development of drug resistance. We identify for the first time the distinctive morphological characterization of endometrial giant macrophage-like cells, called CAMLs, in the context of the presence of circulating tumor cells in patients. Two distinct size-based subtypes of CAMLs, <20 µm (tiny) and >20 µm (giant), were demonstrated. The giant CAMLs have distinctive polymorphic morphologies with mononuclear or fused polynuclear structures, including (1) apoptotic CAMLs; (2) CAML–WBC doublets; (3) conjoined CAMLs; (4) CAML–WBC clusters; and (5) circulating tumor cell–CAML–WBC clusters. In enumerating CAMLs and circulating tumor cells simultaneously, we observed that all circulating tumor cell-positive patients are also positive for CAMLs, in contrast to 55% of patients out of all CAML-positive patients that were found positive for circulating tumor cells.

**Abstract:**

The blood of patients with solid tumors contains circulating tumor-associated cells, including epithelial cells originating from the tumor mass, such as circulating tumor cells (CTCs), or phagocytic myeloid cells (differentiated monocytes), such as circulating cancer-associated macrophage-like cells (CAMLs). We report for the first time the identification and in-depth morphologic characterization of CAMLs in patients with endometrial cancers. We isolated CAMLs by size-based filtration on lithographically fabricated membranes followed by immunofluorescence, using a CD45+/CK 8,18,19+/EpCAM+/CD31+/macrophage-like nuclear morphology, from > 70 patients. Irrespective of the histological and pathological parameters, 98% of patients were positive for CAMLs. Two size-based subtypes of CAMLs, <20 µm (tiny) and >20 µm (giant) CAMLs, of distinctive polymorphic morphologies with mononuclear or fused polynuclear structures in several morphological states were observed, including apoptotic CAMLs, CAML–WBC doublets, conjoined CAMLs, CAML–WBC clusters, and CTC–CAML–WBC clusters. In contrast, CAMLs were absent in patients with non-neoplastic/benign tumors, healthy donors, and leucopaks. Enumerating CTCs simultaneously from the same patient, we observed that CTC-positive patients are positive for CAMLs, while 55% out of all CAML-positive patients were found positive for CTCs. Our study demonstrated for the first time the distinctive morphological characteristics of endometrial CAMLs in the context of the presence of CTCs in patients.

## 1. Introduction

Distant metastasis in solid tumors requires viable tumor cells to travel to the secondary site physically. To form a secondary metastatic colony, epithelial tumor cells of solid tumors undertake a journey that, among many functions, demands the display of specific properties not characteristic of classical epithelial cells. The epithelial tumor cells lose their inherent property of attachment to the ECM (epithelial-mesenchymal transition) prior to the process of intravasation to enter blood/lymphoid circulation and survive in the circulation. Finally, they extravasate to get seeded and colonized, aided by regaining their embryonic epithelioid property by the process of MEC (mesenchymal-epithelial transition). The entire event of the metastasis-associated tumor progression has been postulated to involve various cell types other than tumor cells, which include endothelial progenitor cells, cancer mesenchymal stem cells, hybrid cancer cells, and circulating monocytic cells [1,2,3]. For example, circulating tumor cells (CTCs) can intravasate into the blood/lymphatic system in conjunction with the tumor-associated macrophages (TAMs) via transendothelial migration [4,5]. Thus in addition to CTCs, the peripheral blood of patients with various solid tumors exhibits circulating cancer-associated cells, constituting the elements of liquid biopsy.

Cancer-associated macrophage-like cells (CAMLs) are one of the cellular elements of liquid biopsy in solid tumors. As defined by Adams et al., circulating CAMLs are giant, fused hybrids of multi-nucleated stromal macrophages (of myeloid origin; CD14+/CD11c+) with a sizeable atypical nucleus and a diffuse cytokeratin-positive cytoplasm found in the peripheral blood of patients with solid tumors [6,7]. CAMLs are a distinct population of cells that express epithelial, monocytic, and endothelial protein markers, as identified by Adams et al. in 2014 [6]. The origin of CAMLs was proposed to be related to the disseminated TAMs, which as specialized differentiated macrophages, are known to derive from primary tumors in order to facilitate trans-endothelial migration of CTCs and hence only present in cancer patients.

Although CAMLs are identified in patients with several solid tumors [7,8], detailed morphological characterizations in the context of clinical relevance have been limited to certain types of solid tumors, including patients with breast, pancreatic, prostate [6,9], lung [10], and esophageal cancers [11,12]. CAMLs are also reported in melanoma, renal cell carcinoma, and colorectal cancers [7,13,14].

Here, we report the first in-depth characterization of CAMLs in endometrial cancers. In our cohort, 98% of patients with endometrial cancers were positive for some type of CAMLs in their blood at the time of surgery. Identifying CAMLs from more than 70 patients with endometrial cancers of various histopathological parameters, we identified two distinct size-based types of CAMLs, tiny CAMLs (<20 µM) and giant CAMLs (>20 µM). These two types of CAMLs are characterized by their distinct shapes, nuclear morphologies, expression of epithelial/myeloid markers, and their presence in patients with different histopathological parameters. We also identified several morphological states of CAMLs, including apoptotic CAMLs, CAML–WBC doublets, conjoined CAMLs, CAML–WBC clusters, and CTC–CAML–WBC clusters.

## 2. Methods

### 2.1. Cell Lines and Reagents

Cell lines from endometrial and lung cancers (AN3CA, RL-95-2, and NCI-H441), as well as HUVEC cells, were procured from ATCC and were cultured according to the standard cell culture procedures as per ATCC recommendations. Leucopak and PBMC (peripheral blood mononuclear cells) were procured from Lonza (Lonza Group Ltd., Basel, Switzerland). The CellSieve enumeration kit with either DAPI/CK-FITC/EpCAM-PE/CD45-Cy5 or DAPI/CK-FITC/CD31-PE/CD45-Cy5 was procured from Creatv Microtech (Potomac, MD, USA).

### 2.2. Patients and Blood Collection

Anonymized peripheral blood samples were collected for enumeration of CAMLs and CTC at surgery from patients with endometrial cancers. Informed (IRB approved: Protocol Number Study: 2017.053-100399_ExVivo001) consent for receiving the peripheral blood was obtained from 72 enrolled patients with endometrial cancers. Blood samples were collected in commercially available CellSave collection tubes (Menarini Silicon Biosystems, Bologna, Italy). We included samples from patients with solid tumors at any stage/grade of the disease undergoing surgery/biopsy with or without pre-treatment/history of any previous carcinoma. We did not include any bone-marrow transplant patients or patients with liquid tumors. All experimental protocols were approved by the institutional and/or licensing committee. All methods were carried out in accordance with the relevant guidelines and regulations.

### 2.3. Isolation and Enumeration of CAMLs and CTCs

**Figure 1:**
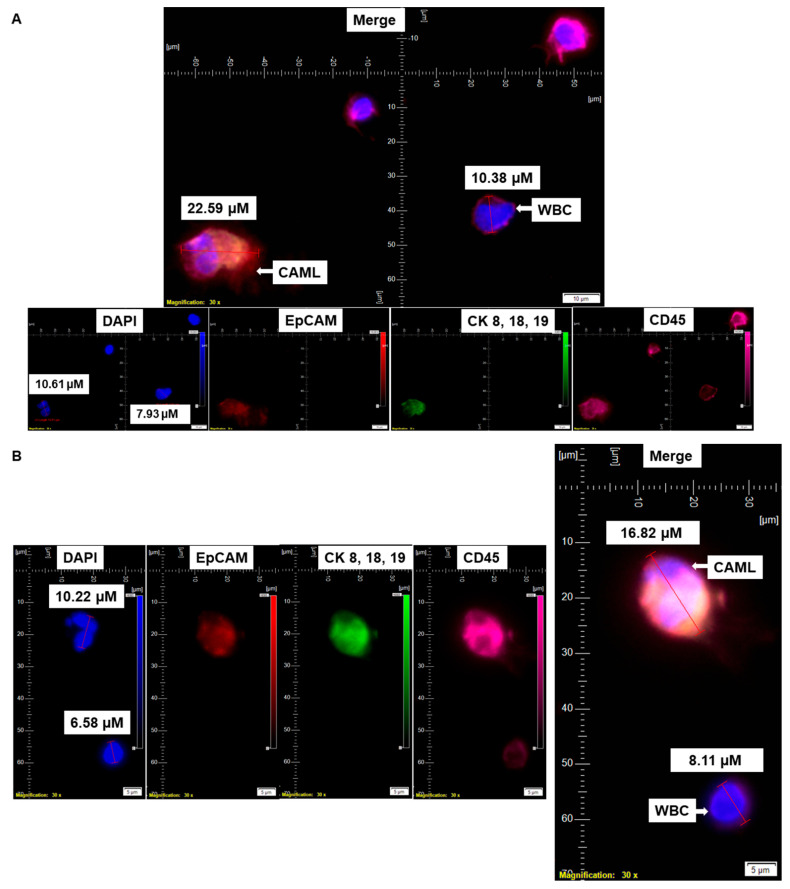

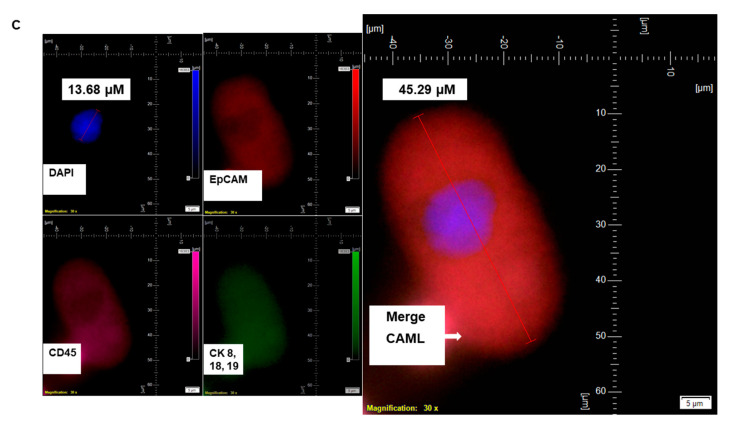
Identification of CD45^+^/CK 8,18,19^+^/EpCAM^+^ CAMLs and their size-based types in the blood of patients with endometrial cancers: Representative pictures of a typical CAML (**A**), a tiny CAML of 16.82 µM (**B**), and a giant CAML of 45.29 µM (**C**) in three patients with endometrial cancers, as enumerated by a CD45-Cy5/CK 8,18,19-FITC/EpCAM-PE immuno-fluorescence kit. WBCs are CD45^+^/CK 8,18,19^−^/EpCAM^−^/DAPI^+^. Pictures were taken with a 60 X oil objective using an Olympus IX71 microscope with DAPI/FITC/PE/CY5 filter sets. Cells were captured on a microfilter and stained with a CellSieve enumeration kit (Creatv Microtech) with DAPI/CK-FITC/EpCAM-PE/CD45-Cy5. The magnification, scale bar, and digital reticle are represented for each photomicrograph. Fluorescence images from the DAPI, FITC, TRITC and Cy5 channels were separated as pictures with a color bar. The fluorescence photomicrographs present the diameters (µm) of a CTC and a representative WBC and their respective DAPI-stained nuclei.

**Figure 2:**
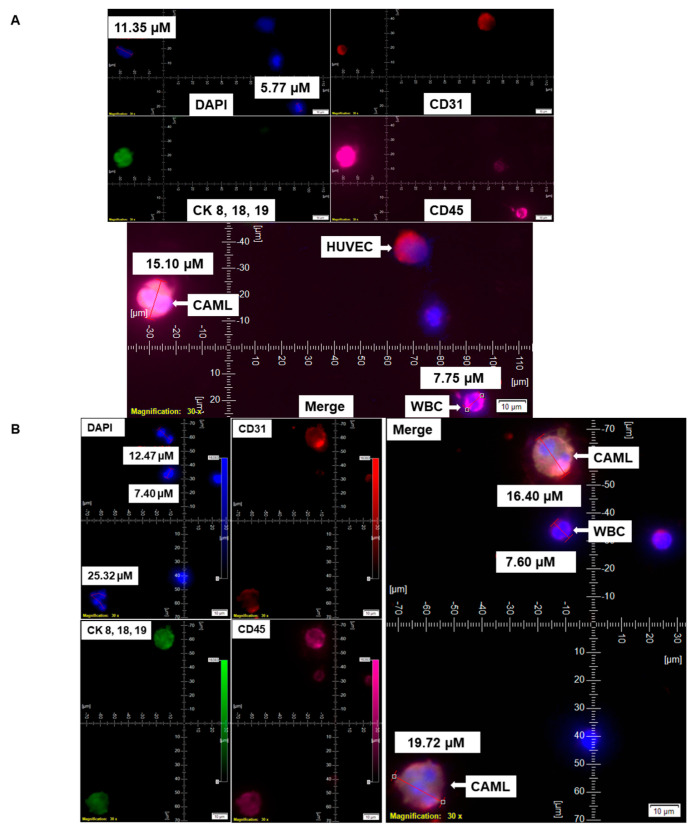
Identification of CD31 positivity of CAMLs in the blood of patients with endometrial cancers. CD31 positivity of CAMLs was tested using CAML tested by a CD45-Cy5/CK 8,18,19-FITC/CD31-PE immuno-fluorescence kit. CD31 positivity was validated in spiked blood samples with HUVEC cells, which are CD31^+^/CK 8,18,19^−^/CD45^−^/DAPI^+^ as the internal positive control (**A**) and in CAMLs from 7.5 mL of patient blood, which are CD31^+^/CK 8,18,19^+^/CD45^+^/DAPI^+^ (**B**) in contrast to CD45^+^/CK 8,18,19^−^/CD31^−^/DAPI^+^ WBCs. Pictures were taken with a 60 X oil objective using an Olympus IX71 microscope with DAPI/FITC/PE/CY5 filter sets. Cells were captured on a microfilter and stained with a CellSieve enumeration kit (Creatv Microtech) with DAPI/CK-FITC/CD31-PE/CD45-Cy5. The magnification, scale bar, and digital reticle are represented for each photomicrograph. Fluorescence images from the DAPI, FITC, TRITC and Cy5 channels were separated as pictures with a color bar. The fluorescence-photomicrographs present the diameters (µm) of a CTC and a representative WBC and their respective DAPI-stained nuclei.

**Figure 3:**
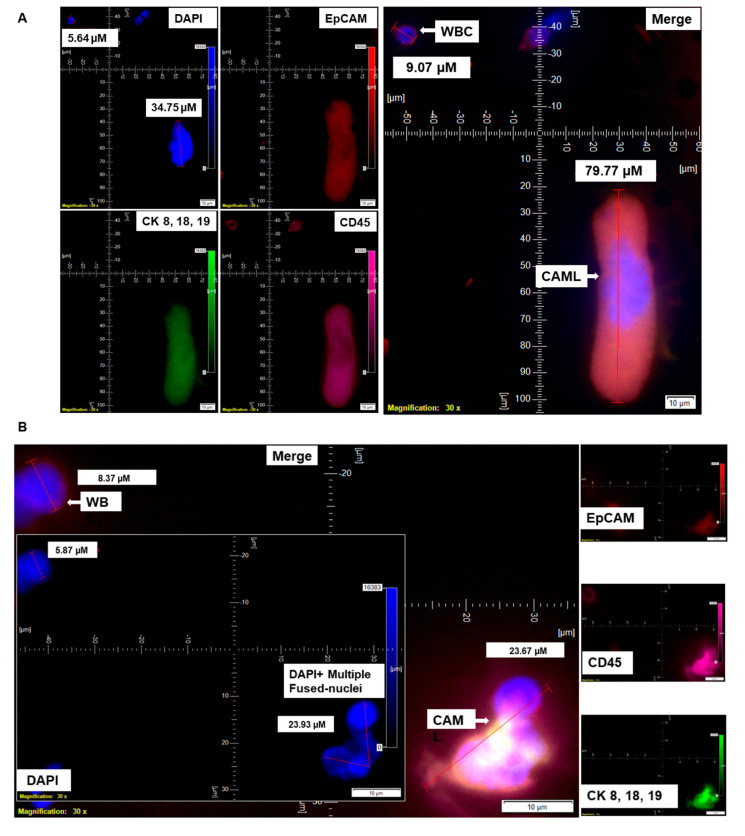

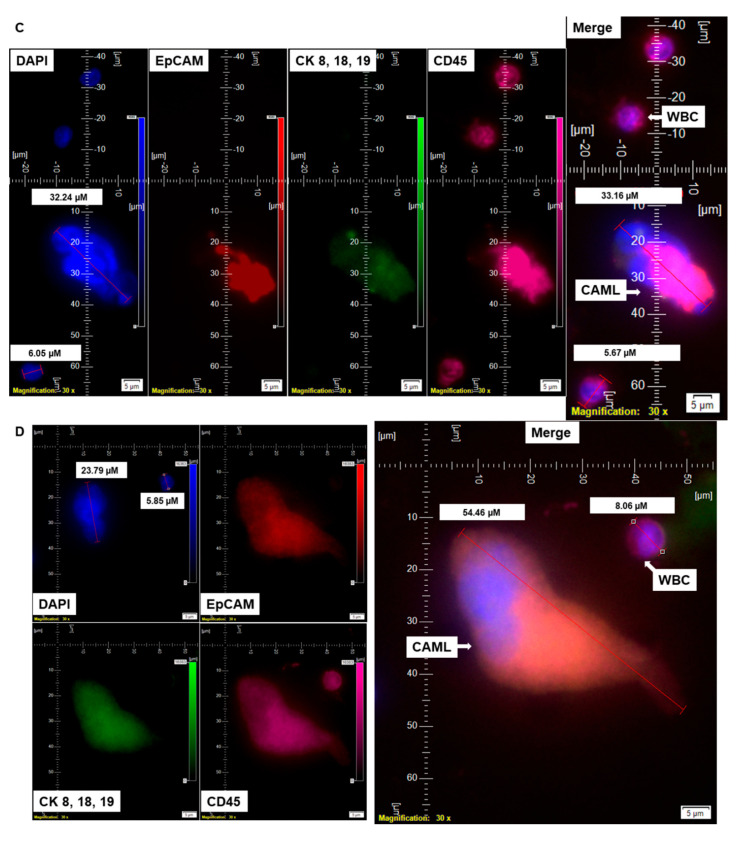

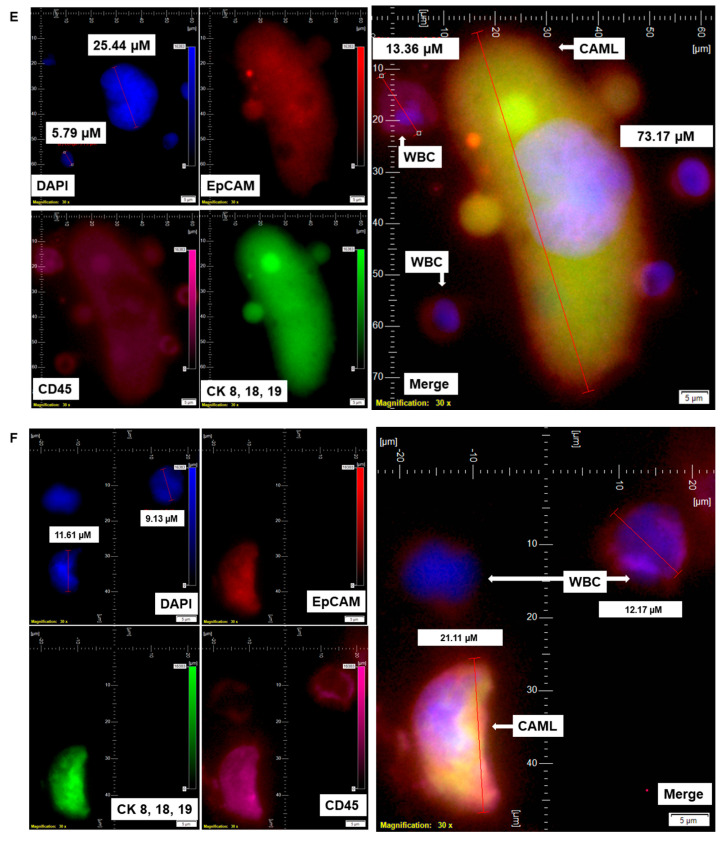
Identification of different shapes of CAMLs in the blood of patients with endometrial cancers. Representative images of 79.77 µM elongated (**A**), 23.67 µM sperm-head-shaped multi-lobed nucleated (**B**), 33.16 µM fused multi-nucleated (**C**), 54.46 µM kidney (**D**), 73.17 µM toy (**E**), and 21.11 µM crescent-moon (**F**) shapes of the giant CAMLs, as identified in the blood of patients with endometrial cancers. The inset in (**B**) shows the DAPI-positive multiple fused nuclei (23.93 µM). Pictures were taken with a 60 X oil objective using an Olympus IX71 microscope with DAPI/FITC/PE/CY5 filter sets. Cells were captured on a microfilter and stained with a CellSieve enumeration kit (Creatv Microtech) with DAPI/CK-FITC/CD45-PE/CD45-Cy5. The magnification, scale bar, and digital reticle are represented for each photomicrograph. Fluorescence images from the DAPI, FITC, TRITC and Cy5 channels were separated as pictures with a color bar. The fluorescence-photomicrographs present the diameters (µm) of a CTC and a representative WBC and their respective DAPI-stained nuclei.

**Figure 4:**
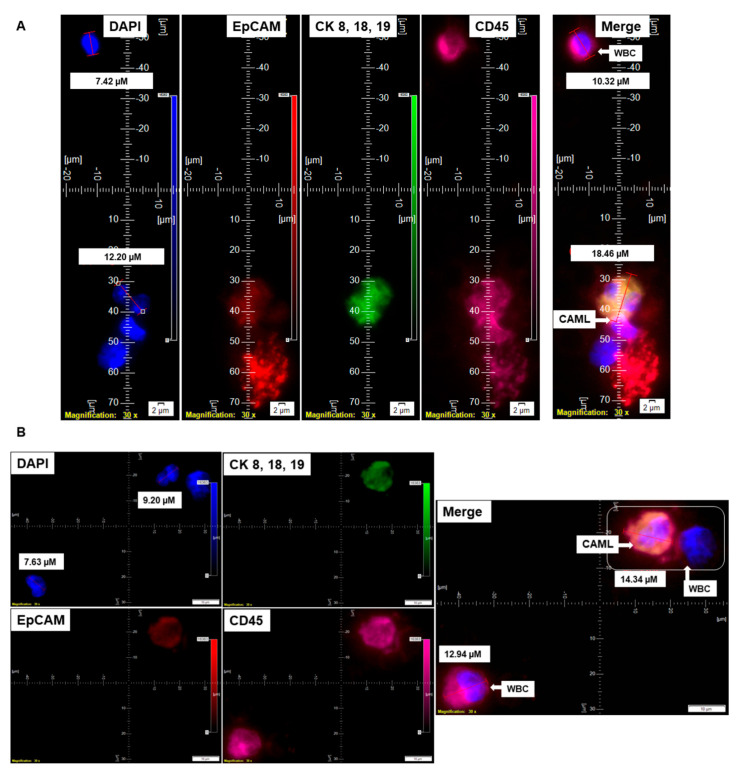

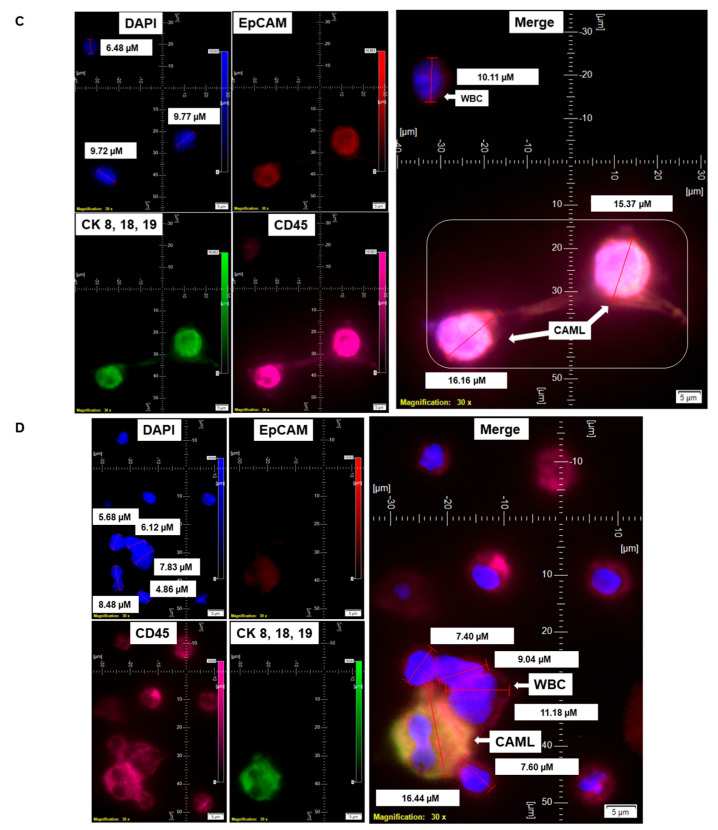
Identification of different states of CAMLs in the blood of patients with endometrial cancers: Representative images of an apoptotic CAML (**A**), CAML–WBC doublet (**B**), conjoined CAML (**C**), and CAML–WBC cluster (**D**), as identified in the blood of patients with endometrial cancers. Pictures were taken with a 60 X oil objective using an Olympus IX71 microscope with DAPI/FITC/PE/CY5 filter sets. Cells were captured on a microfilter and stained with a CellSieve enumeration kit (Creatv Microtech) with DAPI/CK-FITC/EpCAM-PE/CD45-Cy5. The magnification, scale bar, and digital reticle are represented for each photomicrograph. Fluorescence images from the DAPI, FITC, TRITC and Cy5 channels were separated as pictures with a color bar. The fluorescence-photomicrographs present the diameters (µm) of a CTC and a representative WBC and their respective DAPI-stained nuclei.

**Figure 5:**
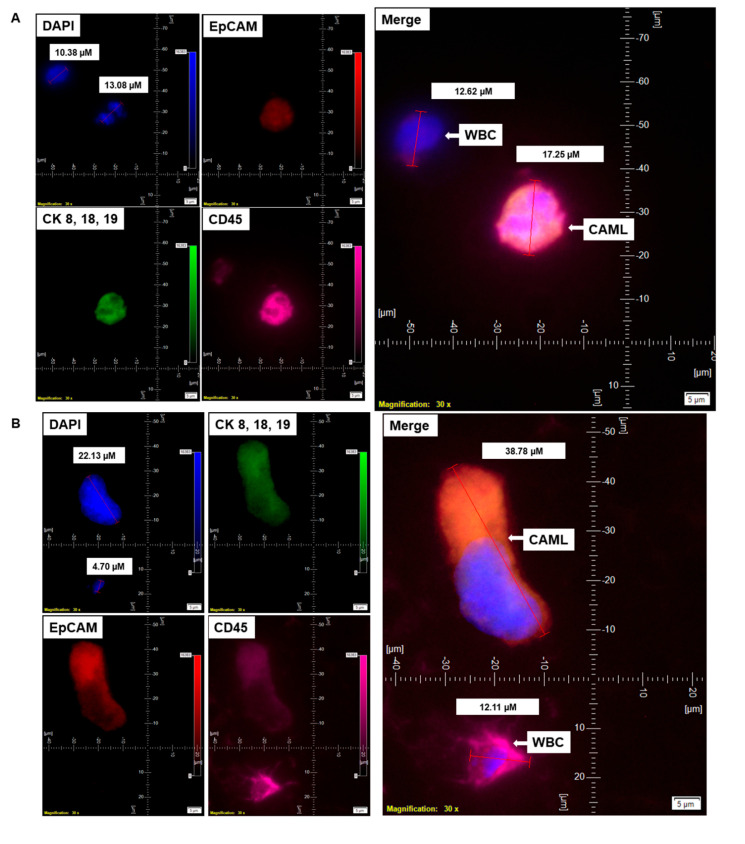

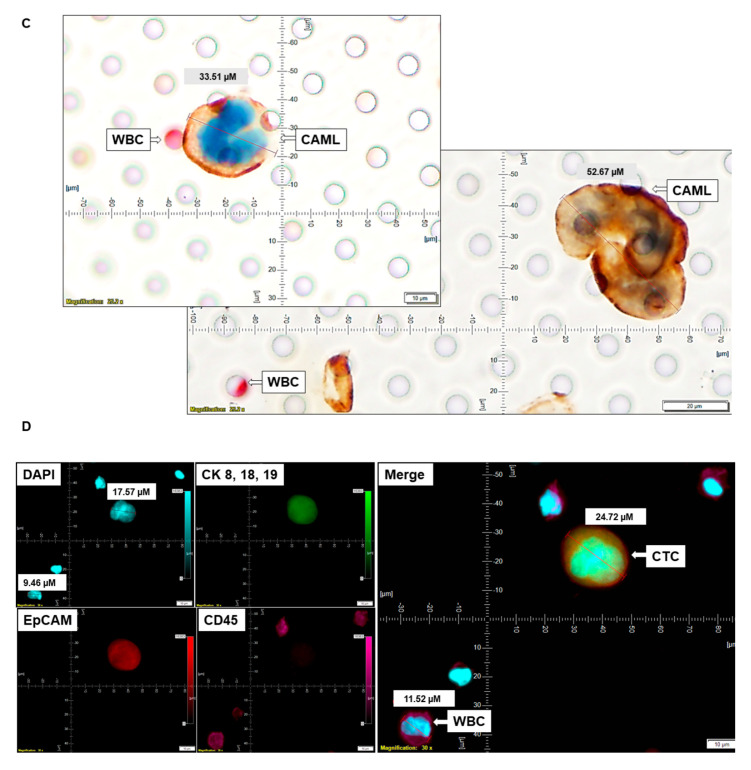

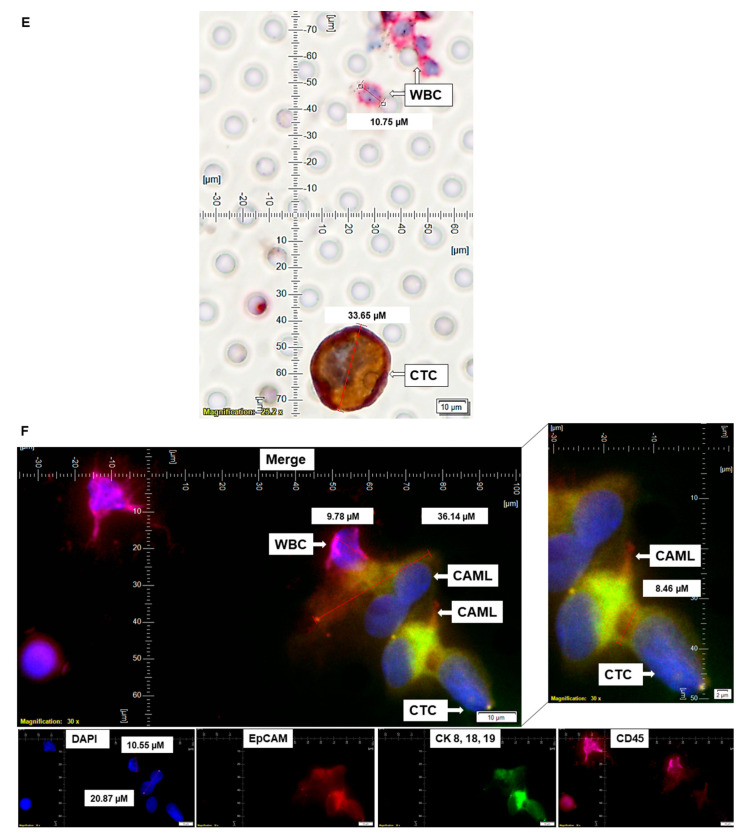
Representative presentation of co-identification of tiny/giant CAMLs and CTC in the blood sample of a patient with endometrioid endometrial adenocarcinoma. A blood sample was used to isolate and enumerate CAMLs and CTCs by triple immunofluorescence and double immunocytochemistry methods. The patient’s blood samples exhibited tiny CAMLs by triple immunofluorescence (**A**), giant CAMLs by immunofluorescence (**B**), tiny (33.51 µM) CAML as well as a giant (52.67 µM) CAML by double immunocytochemistry (**C**), CTC by triple immunofluorescence (**D**), and CTC by double immunocytochemistry (**E**) methods. We identified one CTC–CAML–WBC cluster (**F**) in the same blood sample. (**D**) presents the DAPI signal in cyan to clarify better the "salt–pepper" image of the nucleus of a CTC. The inset in (**F**) presents a larger image of the CAML–CTC to show the cytoplasmic continuum (8.46 µM). Immunofluorescent pictures were taken with a 60 X oil objective using an Olympus IX71 microscope with DAPI/FITC/PE/CY5 filter sets. Cells were captured on a microfilter and stained with a CellSieve enumeration kit (Creatv Microtech) with DAPI/CK-FITC/CD45-PE/CD45-Cy5. The magnification, scale bar, and digital reticle are represented for each photomicrograph. Fluorescence images from the DAPI, FITC, TRITC and Cy5 channels were separated as pictures with a color bar. The fluorescence-photomicrographs present the diameters (µm) of a CTC and a representative WBC and their respective DAPI-stained nuclei. For better clarity, artificial cyan color was used to identify the fluorescence signal from the DAPI. The inset of the merge shows the CK 8,18,19^+^/EpCAM^+^ and 8.46 µM wide inter-cytoplasmic staining between CAML and CTC. ICC pictures were taken with a 40 X objective using an Olympus microscope (model BX43F).

CTC was determined by triple-immunofluorescence (CellSieveTM; Creatv Microtech, Potomac, MD, USA) following isolation using lithographic microfilters as presented elsewhere [15]. In short, 7.5 mL of blood was collected in CellSave collection tubes. CTC and CAMLs were collected on the surface of the microfilter membrane aided by a syringe pump (KD Scientific Legato 110 CMT; Analytical West, Inc., Lebanon, PA, USA) assembled with filter holder assembly (Creatv Microtech; Potomac, MD, USA) and then enumerated using a kit with either DAPI/CK-FITC/EpCAM-PE/CD45-Cy5 or DAPI/CK-FITC/CD31-PE/CD45-Cy5, as procured from Creatv Microtech. Parallel blood samples were spiked with AN3CA/RL-95-2/NCI-H441 tumor cells. Immunofluorescence identified CAMLs parallel to CTCs by immuno-cytochemistry (ICC), as mentioned elsewhere [6,15]. Details of the CellSieveTM CTC Enumeration Standard Kit (Creatv MicroTech, Inc.) are Chroma Part #49000 for DAPI, 49020 for FITC, CellSieve-PE for TRITC/PE, and CellSieve-Cy5 for Cyanine5. We used Cytokeratin 8 and 18 (B22.1 and B23.1) (#818M-94) and Ep-CAM/Epithelial Specific Antigen (Ber-EP4) (248M-94) from Cell Marque. The images were acquired using an Olympus cellSens 1.18 LIFE SCIENCE IMAGING SOFTWARE (OLYMPUS CORPORATION). We used the principle of CD45^−^/CK8,18,19+/EpCAM+/DAPI+ for determining the CTCs (FDA-approved definition of CTC) and CD45+/CK8,18,19+/EpCAM+/DAPI+ for determining CAMLs by the immunofluorescence method. DAPI was used for the evaluation of the nuclear size and morphology. The measurement of the cell and nuclear diameters are presented in the photomicrographs of Figure 1, Figure 2, Figure 3, Figure 4 and Figure 5.

### 2.4. Validation of CAMLs by CD31 Staining

The detection of CAMLs in patients’ blood was validated using CD31 staining. Blood samples were processed, and cells were stained using a kit containing a cocktail of CD45-CK8,18,19-CD31 antibodies. Parallel blood samples were spiked with CD31-positive HUVEC cells. CAMLs were identified as CD45+/CK8,18,19+/CD31+/DAPI+-stained cells in contrast to CD45^−^/CK8,18,19^−^/CD31+/DAPI+-stained HUVEC cells and CD45^−^/CK8,18,19+/CD31^−^/DAPI+-stained tumor cells.

## 3. Results

### 3.1. Identification and Validation of Endometrial CAMLs

Informed consent was obtained from 72 patients with endometrial cancers whose blood samples were received for standardization and detection of CAMLs. Table 1 presents the demographic information of the 72 patients who participated in the study. Table 2A shows the histology of the tumors from the patients with endometrial tumors, and Table 2B presents the pathological parameters of the patients. Endometrial CAMLs were identified in 7.5 mL of a patient’s blood sample at the time of surgery. The identification was based on the CD45+/CK 8,18,19+/CD31+/EpCAM+ immunofluorescent stains with the DAPI+ nuclear morphology of macrophages (Figure 1). Our study showed that the DAPI-stained nuclei of endometrial CAMLs have a characteristic morphology, ranging from a typical central spheroid nucleus to a monocyte-like bi-lobed (U-shaped or kidney-shaped) or multi-lobed nucleus.

We have identified CD45+/CK 8,18,19+/CD31+/EpCAM+/DAPI+-stained CAMLs in more than 98% (63/64) of our tested blood samples in patients with endometrial cancers. Figure 1A presents a CD45+/CK 8,18,19+/EpCAM+/DAPI+-stained 22.59 µM CAML in contrast to a CD45+/CK 8,18,19^−^/EpCAM^−^/DAPI+-stained 10.38 µM WBC from blood of a patient with a Grade 1, Stage IA endometrial endometrioid adenocarcinoma tumor. We tested the CD31 positivity of CAMLs with a parallel validation by CD31-positive HUVEC cells (Figure 2A). Figure 2A shows CD45+/CK 8,18,19+/CD31+/DAPI+-stained CAML, CD45+/CK 8,18,19^−^/CD31^−^/DAPI+-stained WBC, and CD45^−^/CK8,18,19^−^/CD31+/DAPI+-stained HUVEC cells. Figure 2B shows CD45+/CK 8,18,19+/CD31+/DAPI+-stained CAMLs with a lobed nucleus.

### 3.2. Characterization of Distinctive Morphology of Endometrial CAMLs

The nuclear morphology of leukocytes, including monocytes of the immune system, is associated with multiple functions [16,17]. The macrophage polarization state is associated with changes in cell shape [18]. The authors demonstrated that M1 polarization caused cells to flatten into a round, pancake-like shape. In contrast, M2 polarization caused cellular elongation and suggests that cell shape is associated with the macrophage polarization state. In our cohort of 72 patients, we observed two types of endometrial CAMLs based on size: tiny CAMLs, with a diameter of <20 µM (15–20 µM) (Figure 1B), and giant CAMLs, with a diameter of >20 µM (average 45.29 µM approximately) (Figure 1C). All three of the patients (Figure 1A–C) had endometrial endometrioid adenocarcinoma and presented Stage IA and a grade range of 1–2 disease at the time of surgery, with the absence of LVI (lymphovascular invasion) and 6–11% myometrial invasion. As we aligned the presence of giant CAMLs with the histological types of the tumor in patients with endometrial cancers, we observed a predominance of endometrial endometrioid adenocarcinomas for the presence of giant CAMLs. As we observed that tiny endometrial CAMLs are distinctive in their uniformity in both size and shape, in contrast to the giant endometrial CAMLs, which are diverse in shape, we compiled different shapes of the giant endometrial CAMLs from our patient cohort. Figure 3 presents six different shapes of the giant endometrial CAMLs, including a 79.77 µM elongated CAML (Figure 3A), sperm-head-shaped multi-lobed nucleated 23.67 µM CAML (Figure 3B), fused multi-nuclear 33.16 µM CAML (Figure 3C), kidney-shaped 54.46 µM CAML (Figure 3D), toy-shaped engulfing 73.17 µM CAML (Figure 3E), and crescent-shaped 21.11 µM CAML (Figure 3F).

Since it is reported that M2 polarization correlates with an increased degree of cell elongation [18], we quantified the degree of cell elongation as defined by the length of the longest axis divided by the length of the short axis across the cell nucleus [18] in our elongated CAMLs. In our study, the degree of cell elongation ranged from 2.0 to 4.1 µM in elongated giant CAMLs. We identified many giant CAMLs exhibiting elongated shapes in our patient cohort. Although macrophage elongation synergizes with cytokine-mediated stimulation for the M2 polarization phenotype [18], we are yet to know the relationship between elongated CAMLs and the M2 phenotype in our blood samples from patients with endometrial cancers and its clinical relevance.

In addition to different shapes of endometrial CAMLs, we recorded CAMLs in the blood in various states. Figure 4 presents an apoptotic CAML (Figure 4A), CAML–WBC doublet (Figure 4B), conjoined CAML (Figure 4C), and CAML–WBC cluster (Figure 4D) in the blood of patients with endometrial cancers. We observed apoptotic CAML in a cluster of cells with typical nuclear morphology and CD45+/CK 8,18,19+/CD45+ staining in the cytosol. The tiny CAMLs, like giant CAMLs, also appear in doublets with WBCs. However, we only observed conjoined tiny CAMLs in contrast to giant CAMLs. Interestingly, in a differential staining (channel separation) of the conjoined CAMLs, we only observed a cytoplasmic continuum for CK 8,18,19, and CD45 stains but not for the EpCAM stain. A CAML–WBC cluster was found to be more common with tiny CAMLs as compared to giant CAMLs.

### 3.3. Pathological Parameters of Endometrial CAMLs

Table 3 presents the pathological parameters of 63 patients with endometrial cancers bearing different sizes of CAMLs (tiny/giant). We evaluated five pathological parameters, namely, stage, grade, LVI, % of myometrial invasion, and lymph node positivity. First, we evaluated the stage of the disease. Among all patients with tiny CAMLs, 78% had Stage I disease, 4% had Stage II, 12% had Stage III, and 4% had Stage IV disease. Among all patients with tiny and giant CAMLs, 73% had Stage I, 0% had Stage II, 18% had Stage III, and 0% had Stage IV disease. Second, we evaluated the grade of the disease. Among all patients with tiny CAMLs, 55% had Grade 1, 18% had Grade 2, and 20% had Grade 3 disease. Among all patients with tiny and giant CAMLs, 36% had Grade 1, 9% had Grade 2, and 45% had Grade 3 disease. Third, we evaluated the presence of LVI. Among all patients with tiny CAMLs, 20% had positive LVI disease, and 76% had negative LVI disease. Among all patients with tiny and giant CAMLs, 27% had positive LVI disease, and 64% had negative LVI disease. Fourth, we evaluated the % of myometrial invasion of the disease. Among all patients with tiny CAMLs, 45% had a myometrial invasion range of 0–25%, 35% had had a myometrial invasion range of 26–50%, 6% had a myometrial invasion range of 51–75%, and 10% had a myometrial invasion range of 76–100%. Among all patients with tiny and giant CAMLs, 55% had a myometrial invasion range of 0–25%, 27% had had a myometrial invasion range of 26–50%, 9% had a myometrial invasion range of 51–75%, and 0% had a myometrial invasion range of 76–100%. Fifth, we evaluated the node-positivity of the disease. Among all patients with tiny CAMLs, 20% had a positive lymph-node disease, and 65% had a negative lymph-node disease. Among all patients with tiny and giant CAMLs, 18% had a positive lymph-node disease, and 82% had a negative lymph-node disease. We observed that the presence of CAMLs neither correlates with the stage nor grade of the disease.

Table 4 shows that among all patients with Stage I disease, 79% had tiny CAMLs and 17% had tiny and giant CAMLs. Patients with Stage II disease had 100% tiny CAMLS and 0% tiny and giant CAMLs. Patients with Stage III disease had 67% tiny CAMLs and 22% tiny and giant CAMLs. Patients with Stage IV disease had 100% tiny CAMLs and 0% tiny and giant CAMLs. Of all patients with Grade 1 disease, 82% had tiny CAMLs, and 12% had tiny and giant CAMLs. Patients with Grade 2 disease had 90% tiny CAMLS and 10% tiny and giant CAMLs. Patients with Grade 3 disease had 67% tiny CAMLS and 33% tiny and giant CAMLs. Of all positive lymph-node patients, 77% had tiny CAMLs and 15% had tiny and giant CAMLs. Of all negative lymph-node patients, 76% had tiny CAMLs, and 21% had tiny and giant CAMLs. Of all positive LVI patients, 71% had tiny CAMLs, and 21% had tiny and giant CAMLs. Of all negative LVI patients, 80% had tiny CAMLs, and 15% had tiny and giant CAMLs. Of all patients with positive myometrial invasion, 77% had tiny CAMLs, and 17% had tiny and giant CAMLs. Of all patients with negative myometrial invasion, 88% had tiny CAMLs, and 12% had tiny and giant CAMLs. Interrogating the pathological parameters, we observed that the skewness of the value could be explained by the distribution of the patients in different pathological parameters, as presented in Table 2B.

Table 5 presents the distribution of CAMLs in different histological types of endometrial cancers in our cohort. Endometrioid adenocarcinoma is our cohort’s overtly predominant histology type of endometrial cancer. With this histology type, most of our patients presented with lower stages and grades of the disease. The reason for this distribution cannot be explained currently. However, demographics and organ type are two factors that can be speculated to justify such a pattern of distribution. Within this group of endometrioid adenocarcinomas, 83% of patients exhibited tiny CAMLs. The highest % (47%) of patients with endometrioid adenocarcinoma presented a few CAMLs. Abundant numbers of tiny CAMLs were presented in 15% of the patients.

### 3.4. Co-Presence of the Endometrial CAMLs with CTC

CAMLs (either tiny or giant) were identified in close to 100% of the tested blood samples from patients. CAMLs are one of the cells that can be identified in liquid biopsies in solid tumors [6]. In our cohort, we tested the presence of CAMLs vis-a-vis the presence of CTC in the blood of patients with endometrial cancers. For this purpose, we enumerated both CAMLs and CTCs in the blood of patients with endometrial cancers by parallel triple immunofluorescence as well as by double immunocytochemistry. Figure 5 shows a representative photomicrograph of both tiny and giant CAML identified by triple immunofluorescence as well as by double immunocytochemistry from the blood samples from a patient with endometrial endometrioid adenocarcinoma with Stage IA and Grade 1 disease at the time of surgery.

We identified CTC in the same patient by triple immunofluorescence (Figure 5A,B,D) and double immunocytochemistry (Figure 5C,E). We identified a single CTC giant CAML–WBC cluster (Figure 5F) in the same blood sample. The cluster consisted of a CD45+/CK 8,18,19-/EpCAM-/DAPI+ WBC, two CD45+/CK 8,18,19+/EpCAM+/DAPI+ CAMLs, and a CD45-/CK 8,18,19+/EpCAM+/DAPI+ CTC, as stained by immunofluorescence. The cluster of a CTC giant CAML–WBC cluster was found to have a CK 8,18,19/EpCAM staining continuum between the CD45+/CK 8,18,19+/EpCAM+ CAML and CD45-/CK 8,18,19+/EpCAM+ CTC.

**Figure 6:**
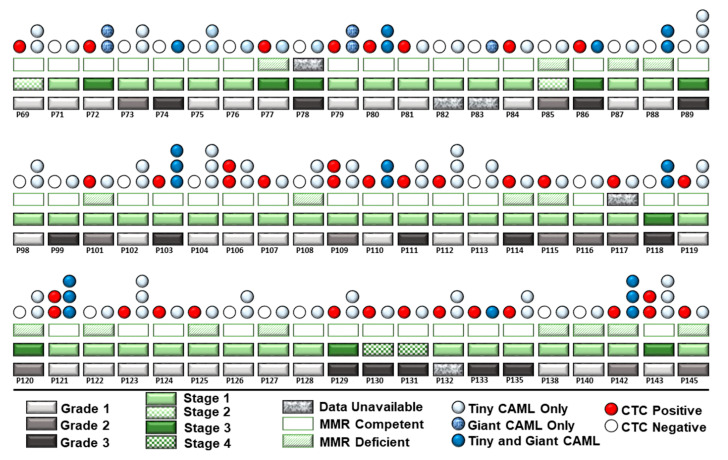
Composite representation of the presence of the size-based types of CAMLs with the histological stages, grades, MMR status, and CTC status in 60 consecutive patients with endometrial tumors: light-grey rectangles for Grade 1, dark-grey rectangles for Grade 2, and black rectangles for Grade 3; light-green rectangles for Stage I; light-green and white checkerboard rectangles for Stage II; dark-green rectangles for stage III; and dark-green and white checkerboard rectangles for Stage IV; green open rectangle for MMR competent, green-white hatched for MMR deficient; and granite rectangles for unavailable data; light-blue circle for tiny CAML, denim blue-circle for giant CAMLs, and dark-blue circles for tiny plus giant CAMLs; red circles represent CTC-positive, and open-circles represent CTC-negative patients. Quantitatively, one blue circle represents a few, two vertical circles represent frequent, and three vertical circles represent abundant CAMLs in the blood. Each patient is represented in a column.

We evaluated the co-expression of CAMLs and CTCs in our patient cohort. A pictorial representation of grades, stages, MMR status in the context of the presence of CTC, and types of CAMLs in Figure 6 show that out of 60 consecutive patients in which both CTCs and CAMLs were tested, more than 55% (33 out of 60) had CTCs. No correlation was observed between MMR status and CAMLs. Out of 33 patients with the presence of both CTCs and CAMLs, 23 (69%) had CTCs with the concomitant tiny CAMLs, and 10 (30%) patients had CTCs with the concomitant tiny/giant CAMLs. Out of 23 patients, 12 patients had a CTC with the concomitant few tiny CAMLs, and 11 patients out of 23 had frequent/abundant CAMLs concomitant with CTCs. We have not observed a correlation between CTC and CAMLs in the blood of patients with endometrial cancers, probably indicating functional exclusivity.

The distribution of giant CAMLs, when coupled with the presence of CTC in our study, showed no association with pathological parameters such as grade, stage, LVI, or % of myometrial invasion. The 79.77 µM elongated giant CAML with more than 1 DAPI+ fused nuclei from the blood sample of the CTC-positive patient with endometrioid adenocarcinoma (Figure 3A) was presented with a disease of Grade 1, Stage IA, absence of LVI, and 9% myometrial invasion. In contrast, the 21.11 µM crescent-shaped giant CAML from the blood sample of the CTC-positive patient with carcinosarcoma (Figure 3F) had a disease of Grade 3, Stage IIIC1, presence of LVI, and 72% myometrial invasion.

## 4. Discussion

CAMLs are specialized polyploid myeloid cells transiting in the circulation of patients with various types of solid malignancies [6,19]. We report the identification of CD45+/CK 8,18,19+/EpCAM+/CD31+/DAPI+ CAMLs in the blood of patients with endometrial cancers. The differential staining of the cells in the liquid biopsy of patients showed distinctive markers, as presented in Table 6. Specific sets of markers used to detect CAMLs, CTCs, WBCs, spiked tumor cells, and endothelial cells by immunofluorescence presented in the table indicate their distinctive expression of marker proteins. We report that size-based filtration and capture can rapidly and efficiently isolate multiple varieties of circulating tumor-associated cells, including CAMLs and CTCs, from peripheral blood of patients with endometrial cancers. We tested the specificity of CAMLs using their CD31 positivity. Adams et al. reported that CAMLs are 96% positive for the endothelial marker, CD31, in addition to their 89% positivity for the epithelial markers, including cytokeratin 8, 18, and 19, and a 45% positivity for EpCAM [20]. We used HUVEC cells as the validation control for CD31.

Circulating cells in the peripheral blood of patients with solid tumors can be of several types, including circulating tumor cells, modified blood cells, plasma cells, fused cancer cells, or giant macrophage-like cells (CAMLs). Circulating cancer-associated cell subtypes includes (1) traditional CTCs (EpCAM+/CK 8,18,19+/CD45^−^ with a DAPI+ ≥8 µM “salt–pepper” nucleus); (2) CTC clusters (≥2 EpCAM+/CK 8,18,19+/CD45^−^/DAPI+ CTCs in aggregate); (3) apoptotic CTCs (lesser DAPI fluorescence intensity compared to a CTC with dotted cytoplasmic CK 8,18,19 stain); (4) CTC debris (EpCAM+/CK 8,18,19+/CD45-/DAPI, <4 µM); (5) EMT CTCs (EpCAM±/CK 8,18,19+/EMT vimentin+, N-Cadherin+); (6) stem-cell CTCs (EpCAM+/CK 8,18,19+/CD133+/CD44+/CD24^−^/ALDH1+); (7) PD-L1-CTCs (EpCAM+/CK 8,18,19+/PD-L1+/CD45^−^/DAPI+); and (8) macrophage–tumor fusion cells (EpCAM+/CK+/CD14+/CD45+, ≥30 µM with diffuse/nonfilamentous cytoplasmic CK 8,18,19+ staining pattern with a ≥1 DAPI+ nucleus and polymorph cell shape). CAMLs belong to the category of cancer-associated circulating extra-CTC cells characterized by distinctly polymorphic, mononuclear/polynuclear, and polymorphonuclear (syn- or heterokaryon) features with dual epithelial and macrophage/myeloid marker phenotypes. They collectively comprise the components of blood-based biopsies, i.e., "liquid" biopsies. Thus, CAMLs are components of "liquid" biopsies. In our study, we identified a novel subset of CAML, tiny CAMLs (15–20 µM), for the first time in the blood of endometrial patients. We also observed that the distribution of tiny CAMLs is not specific for any stage/grade/histological type of the disease. These tiny CAMLs can be accompanied by the classical giant (≥30 µM) CAMLs. Although it has been reported that macrophages adopt different geometries in vivo; how these changes in cell shape might feed back into regulating their functional phenotype has not been delineated [18]. The cell shape, however, plays a vital role in the modulation of the macrophages’ phenotypic polarization [18]. The study reported that macrophages exhibit different degrees of elongation when stimulated toward the M1 or M2 phenotypes with cytokines in vitro. The elongation of cells induced polarization to an M2 phenotype. Endometrial CAMLs, tiny and giant, presented two characteristic patterns for our study for CK 8,18,19+ stains. We demonstrated both a diffuse CK 8,18,19+ stain (Figure 1C and Figure 3A,D,E) and a more condensed asymmetrical expression of the protein in CAMLs (Figure 1A, Figure 2A,B, Figure 3B,F and Figure 4A–D). Endometrial CAMLs exhibited diverse mononuclear/multi-lobed or polynuclear/fused nuclei (Figure 3B,C) of polymorphic cell shapes. The range of sizes, shapes of cells, and their nuclei raise the possibility of cell fusion to explain the origin and characterization of CAMLs in endometrial cancers.

Reports of circulating cancer-associated cells bearing both epithelial and macrophage/myeloid phenotypes and associated genetic evidence indicate that cell fusion has a critical role in the progression of the disease in patients [21]. There vehave been genetic evidence for the presence of fusion cells in cancer patients, and tumor cell fusion with immune cells, specifically with macrophages, is reported to be associated with the development of metastasis by acquiring features such as genetic/epigenetic heterogeneity, immune tolerance, and chemotherapeutic resistance [7]. Macrophages with high fusogenic potential can fuse into tumor cells [7,22]. In breast cancers, macrophage traits in cancer cells are reported to be induced by macrophage–cancer cell fusion, which cannot be explained by paracrine cellular interaction [23]. Strikingly, patient-derived circulating macrophage-tumor cell fusions were reported to present M2 macrophage phenotypes in PDAC (pancreatic ductal adenocarcinoma) and melanoma [24,25]. Indeed, intercellular connections between tumor cells and macrophages have been reported, leading to partial fusion via membrane protrusions [26]. It is proposed that open-ended cellular projections, called "Tunneling NanoTubes" [26,27], displaying diameters ranging from 20 to 500 nm, allowing a direct physical connection between the cytoplasms of two or more cells that belong to different cell types (heterotypic) or the same cell type (homotypic), play a role in tumor cell interactions with macrophages in various solid tumors [28,29]. We observed a CTC giant CAML–WBC cluster in the blood of patients with endometrial cancers. The inset of the merge in Figure 5F showed a CK 8,18,19+/EpCAM+ cytoplasmic extension between the CAML and CTC of the cluster. Interestingly, the structure was CD45 negative (Figure 5F inset). It will be worthwhile to test M2 markers for these CTC giant CAML–WBC clusters in the context of clinical relevance.

We also observed that the morphology of the DAPI-stained nucleus of endometrial CAMLs ranged from a typical central spheroid nucleus to a monocyte-like bi-lobed (U-shaped or kidney-shaped) or multi-lobed nucleus. Interestingly, nuclear morphology determines two important functions of a cell, including transcriptional activity and flexibility of movement. Indeed, leukocytes of the immune system have lobed nuclei, which are known to impart their flexibility and migration [16]. Cellular monocytes present a lobed nucleus, with the lobes being larger and fewer than circulating neutrophils. Monocytes are also flexible enough to enter tissues, after which they differentiate into various other cell types, including macrophages. Since macrophages remain functionally plastic throughout their lifetime, it is reported that they can change between roles with relative ease [17], thereby, via their readily deformable nucleus, facilitating transcriptional regulation [16]. The diversity of the nuclear morphology in endometrial CAMLs identified for the first time in our study strongly indicates their functional relevance in the context of their movement and regulation of gene expression.

In the organ-type of solid tumors where CAMLs have been identified so far, their presence has been reported in every stage of the disease [6,19]. In line with this report, we observed that CAMLs were correlated neither to the stage nor grade of disease in endometrial cancers (Table 3 and Table 4). Table 3 presents the pathological parameters of all patients with endometrial cancers, bearing two identifiable sizes of CAMLs—tiny and giant. In turn, Table 4 tabulates the CAMLs present in the blood of patients, as categorized by stage, grade, lymph node positivity, LVI, and myometrial invasion of the tumors. CAML+ve patients predominantly present endometrioid adenocarcinoma histology (Table 5). However, it cannot be viewed as any inference owing to the fact that (1) this histology is the most common histology in endometrial cancers; and/or (2) our cohort had the highest number of patients who have a disease of this histology. The primary limitation of our study is that our cohort of patients with endometrial cancers is skewed demographically, histologically, and in presenting the stage and grade of the disease. Most of our patients are Caucasian with endometrioid endometrial adenocarcinomas of a low stage and grade.

Macrophages contribute to the various stages of tumor progression, from initiation to formation of distant metastasis [30]. The relevance of a CAML–WBC cluster can be viewed from the perspective of the wheels of macrophage activation, such as activated M2 or M2-like macrophages under pathological conditions [17]. With their remarkable plasticity (priming, polarized activation, training, and tolerance), macrophages change their functions in response to environmental cues, giving rise to different populations of cells with distinct functions. Hence, macrophages are grouped based on various homeostatic activities—host defense, wound healing, and immune regulation—in normal and disease conditions [30]. Considering the role of T-cell-mediated modification of macrophages in cancers, the relevance of CAML–WBC clusters in the blood samples of patients with endometrial cancers remains to be seen.

Fusion cells hold a critical role in being a key component in cancer progression, metastasis, and drug resistance [7]; indeed, their study could evolve as a diagnostic and therapeutic target in precision medicine for cancers. CAMLs could be viewed as a mirror-room peephole of a tumor’s evolution, response/resistance to therapy, and progression. Our data on CAMLs raise a few unresolved questions about endometrial cancers. *Considering their size, particularly giant CAMLs, if they originate from tumor tissue, then what is their mode and route from a distant primary tumor? Expressing markers for both CTCs and macrophages, how close are CAMLs, both tiny and giant, to TAM or M2? Are they a product of a fusion of a cancer cell/CTC and macrophage (M2)? What is their contextual relation with WBCs/T-cells in circulation?* Although our study cannot answer such questions at present, interrogations will be crucial in determining the functions and clinical relevance of CAMLs in endometrial cancers. Macrophage fusion hybridization with tumor cells [31,32] is reported to have a distinct role in disease progression and metastasis [7,33]. Indeed, in NSCLCs, circulating giant tumor–macrophage fusion cells are reported to be an independent prognosticator [34]. M2 macrophages have potential pro-tumor functions by virtue of their role in immune evasion [35,36]. Since CAMLs exhibit macrophage markers in addition to epithelial markers [6,7], it can be speculated that CAMLs may have a role in immunotherapy resistance. Cellular liquid biomarkers present the potential to complement the diagnostic interpretation of patients and empower the management of a disease. Circulating tumor–macrophage fusion cells and CTC have been reported to complement NSCLC screening [37]. Future studies will clarify the functional and clinical relevance of CAMLs in endometrial cancers.

## 5. Conclusions

Our data confirm the presence of CAMLs in the peripheral blood of patients with endometrial tumors. The CAMLs present in the blood of these patients are morphologically classified into tiny and giant based on their size. Giant CAMLs present a wide range of morphologies and forms, including apoptotic and in doublet/clusters with WBC or CTCs. In enumerating CTCs and CAMLs simultaneously from the blood of the same patient, we observed that all CTC-positive patients are positive for CAMLs in contrast to 55% out of all CAML-positive patients who were found positive for CTCs. Our study demonstrates for the first time the distinctive morphological characteristics of endometrial CAMLs in the context of the presence of CTC in the patients. Our data indicate a need for an in-depth study on the clinical relevance of CAMLs in the context of CTC in endometrial cancers.

## 6. Patents

The study presented in the MS is part of a patent application (United States Patent and Trademark Office; Application number 16/875,910).

## Figures and Tables

**Table 1:** Demographic information of the 72 patients included in the study.

Patient Information	Values * (Values Are % *(n)* Unless Otherwise Specified)*(n = 72)*
**Age at Surgery (years), median (range)**	65 (*43–84*)
**ECOG performance status**	
0	79% (*57*)
1	15% (*11*)
2	0% (*0*)
3	4% (*3*)
4 to 5	0% (*0*)
Not Available	1% (*1*)
**Ethnicity**	
African	1% (*1*)
Caucasian	99% (*71*)
**Smoking History**	
Yes	22% (*16*)
No	78% (*56*)
**BMI, median (range)**	35.55 (*21.9–62.7*)

* Includes blood samples from seven patients used for standardization and one blood sample from a patient that was not tested.

**Table 2:** (**A**) Histology of the tumors from the patients with endometrial cancers. (**B**) Stage, grade, lymph node positivity, lymphovascular invasion (lvi), and myometrial invasion of tumors from patients with endometrial cancers.

(A)				
Histology *			**Values** (Values are listed as % (*n*)) (*n* = *72*)	
**Benign endometrial polyp**			3% (*2*)	
**Carcinosarcoma**			4% (*3*)	
**Carcinosarcoma with high-grade serous carcinoma and rhabdomyoma sarcomatous differentiation**			1% (*1*)	
**Complex atypical hyperplasia**			3% (*2*)	
**Endometrioid adenocarcinoma**			76% (*55*)	
**High-grade endometrial adenocarcinoma**			1% (*1*)	
**High-grade papillary serous carcinoma**			1% (*1*)	
**High-grade mixed endometrial adenocarcinoma, clear cell and serous 50% each**			1% (*1*)	
**High-grade serous endometrial adenocarcinoma**			4% (*3*)	
**Mixed-cell carcinoma, high-grade (10% serous carcinoma, 90% endometrioid carcinoma)**			1% (*1*)	
**Mixed-cell carcinoma, high-grade (90% high-grade serous carcinoma, 10% endometrioid adenocarcinoma)**			1% (*1*)	
**Residual carcinosarcoma**			1% (*1*)	
**(B)**				
Stage *				
I	II	III	IV	NA
76% (*48*)	3% (*2*)	14% (*9*)	3% (*2*)	3% (*2*)
Grade *				
1	2	3		NA
52% (*33*)	16% (*10*)	24% (*15*)		8% (*5*)
Lymph Node Positivity *				
Yes	No		NA	
21% (*13*)	67% (*42*)		13% (*8*)	
Lymphovascular Invasion (LVI) *				
Yes	No		NA	
22% (*14*)	73% (*46*)		5% (*3*)	
Myometrial Invasion *				
Yes	No		NA	
82% (*52*)	13% (*8*)		5% (*3*)	

* Values are % (*n*) (*n* = *63*). NA = not available.

**Table 3:** Pathological parameters of 63 patients with endometrial cancers bearing different sizes of CAMLs (tiny/giant).

Pathological Parameters	% of Patients with Tiny CAMLs only (*n* = *49*)	% of Patients with Both Tiny and Giant CAMLs * (*n* = *11*)
**Stage I**	78% (*38*)	73% (*8*)
**Stage II**	4% (*2*)	0% (*0*)
**Stage III**	12% (*6*)	18% (*2*)
**Stage IV**	4% (*2*)	0% (*0*)
**No Stage Determined**	2% (*1*)	9% (*1*)
		
**Grade 1**	55% (*27*)	36% (*4*)
**Grade 2**	18% (*9*)	9% (*1*)
**Grade 3**	20% (*10*)	45% (*5*)
**No Grade Determined**	6% (*3*)	9% (*1*)
		
**Lymphovascular Invasion Present**	20% (*10*)	27% (*3*)
**Lymphovascular Invasion Absent**	76% (*37*)	64% (*7*)
		
**Myometrial Invasion 0–25%**	45% (*22*)	55% (*6*)
**Myometrial Invasion 26–50%**	35% (*17*)	27% (*3*)
**Myometrial Invasion 51–75%**	6% (*3*)	9% (*1*)
**Myometrial Invasion 76–100%**	10% (*5*)	0% (*0*)
		
**Lymph Node-Positive**	20% (*10*)	18% (*2*)
**Lymph Node-Negative**	65% (*32*)	82% (*9*)

* Three patients exhibited only giant CAMLs.

**Table 4:** Distribution of subtypes of CAMLs in the blood of patients with endometrial cancers with different stages, grades, lymph node positivity, lymphovascular invasion (lvi), and myometrial invasion.

Stage *					
Size of CAMLs	I	II	III	IV	NA
Tiny Only	79% (*38*)	100% (*2*)	67% (*6*)	100% (*2*)	50% (*1*)
Tiny + Giant	17% (*8*)	0% (*0*)	22% (*2*)	0% (*0*)	50% (*1*)
Grade *					
Size of CAMLs	1	2	3		NA
Tiny Only	82% (*27*)	90% (*9*)	67% (*10*)		60% (*3*)
Tiny + Giant	12% (*4*)	10% (*1*)	33% (*5*)		20% (*1*)
Lymph Node Positivity *					
Size of CAMLs	Yes		No		NA
Tiny Only	77% (*10*)		76% (*32*)		88% (*7*)
Tiny + Giant	15% (*2*)		21% (*9*)		0% (*0*)
Lymphovascular Invasion (LVI) *					
Size of CAMLs	Yes		No		NA
Tiny Only	71% (*10*)		80% (*37*)		67% (*2*)
Tiny + Giant	21% (*3*)		15% (*7*)		33% (*1*)
Myometrial Invasion *					
Size of CAMLs	Yes		No		NA
Tiny Only	77% (*40*)		88% (*7*)		67% (*2*)
Tiny + Giant	17% (*9*)		12% (*1*)		33% (*1*)

* Values are % (*n*) (*n* = *63*). NA = not available.

**Table 5:** Distribution of CAMLs in different histological types of endometrial cancers in our cohort.

Histology of Tumors of the Patients with Endometrial Cancers	Total # of Patients (*n* = 62)	Types of CAMLs in Different Histological Types of Endometrial Cancers		Semiquantification of Tiny CAMLs in Different Histological Types of Endometrial Cancers		
		Tiny + Giant	Tiny Only	Tiny CAML Few	Tiny CAML Frequent	Tiny CAML Abundant
		% (*n*)		% *(n)*		
Carcinosarcoma	3	33% (*1*)	67% (*2*)	67% (*2*)	33% (*1*)	0% (*0*)
Carcinosarcoma with high-grade serous carcinoma and rhabdomyoma sarcomatous differentiation	1	0% (*0*)	100% (*1*)	0% (*0*)	0% (*0*)	100% (*1*)
Complex atypical hyperplasia	2	50% (*1*)	50% (*1*)	100% (*2*)	0% (*0*)	0% (*0*)
Endometrioid adenocarcinoma	47	13% (*6*) *	83% (*39*)	47% (*22*)	34% (*16*)	15% (*7*)
High-grade endometrial adenocarcinoma	1	100% (*1*)	0% (*0*)	100% (*1*)	0% (*0*)	0% (*0*)
High-grade papillary serous carcinoma	1	0% (*0*)	100% (*1*)	100% (*1*)	0% (*0*)	0% (*0*)
High-grade mixed endometrial adenocarcinoma, clear cell and serous 50% each	1	0% (*0*)	100% (*1*)	100% (*1*)	0% (*0*)	0% (*0*)
High-grade serous endometrial adenocarcinoma	3	33% (*1*)	67% (*2*)	33% (*1*)	67% (*2*)	0% (*0*)
Mixed-cell carcinoma, high grade (10% serous, 90% endometrioid)	1	100% (*1*)	0% (*0*)	100% (*1*)	0% (*0*)	0% (*0*)
Mixed-cell carcinoma, high grade (90% serous, 10% endometrioid)	1	0% (*0*)	100% (*1*)	0% (*0*)	100% (*1*)	0% (*0*)
Residual carcinosarcoma	1	0% (*0*) *	0% (*0*)	0% (*0*)	0% (*0*)	0% (*0*)

* = This histology also had patients with only Giant CAMLs.

**Table 6:** Specific sets of markers were used to detect CAMLs, CTCs, WBCs, spiked tumor cells, and endothelial cells by immunofluorescence.

Name of Marker	CK8,18,19	EpCAM	CD45	CD31	DAPI
Specific For	Epithelial Marker: Cytoskeleton	Epithelial Marker: Adhesion Molecule	Leucocyte Common Antigen	Endothelial Cell Marker	Nuclear Morphology
Filter	FITC	TRITC/PE	Cy5	TRITC/PE	DAPI
Color (Natural/Artificial)	Green	Red	Magenta	Red	Blue/Cyan
**CTC**	Positive	Positive	Negative	Negative	Positive
**Spiked Tumor Cell (NCI-H441)**	Positive	Positive	Negative	Negative	Positive
**WBC**	Negative	Negative	Positive	Negative	Positive
**CAML**	Positive	Positive	Positive	Positive	Positive
**Endothelial Cell (HUVEC)**	Negative/±	Negative	Negative	Positive	Positive

## Data Availability

All data can be found in the text.
